# Hepatitis D Virus: Enigmas and Gaps of Knowledge

**DOI:** 10.3390/v18020244

**Published:** 2026-02-14

**Authors:** Flor H. Pujol, Rossana Celeste Jaspe, Armando Andres Roca Suarez, Enkhtuul Batbold, Fabien Zoulim, Barbara Testoni, Isabelle Chemin

**Affiliations:** 1 Laboratorio de Virología Molecular, Centro de Microbiología y Biología Celular, Instituto Venezolano de Investigaciones Científicas (IVIC), Caracas 1020A, Venezuela; rossanajaspesec@gmail.com; 2 INSERM Unité Mixte de Recherche 1350, PaThLiv IHU EVEREST, Université Claude-Bernard Lyon 1, 69003 Lyon, France; armando-andres.roca-suarez@inserm.fr (A.A.R.S.); batbold.enkhtuul@inserm.fr (E.B.); fabien.zoulim@inserm.fr (F.Z.); barbara.testoni@inserm.fr (B.T.); isabelle.chemin@inserm.fr (I.C.)

**Keywords:** hepatitis D virus, *Deltavirus*, replication, hepatocellular carcinoma, eradication

## Abstract

Hepatitis D virus (HDV) is a very peculiar virus that shares many characteristics with plant viroids. One of its unique characteristics is the requirement for the presence of a helper virus for its replication, and in particular enveloping its virion, a role often played by the hepatitis B virus (HBV). Infection with HDV is frequently associated with more severe disease, which may present with fulminant hepatitis or a more rapid progression to cirrhosis and hepatocellular carcinoma (HCC), when compared to HBV mono-infection. HDV exhibits many peculiarities and enigmas, which have led to it being considered a neglected virus. This review aims to identify the most important gaps in knowledge and peculiarities in the study of this enigmatic virus, from virology to clinical implications.

## 1. Introduction

**Figure 1:**
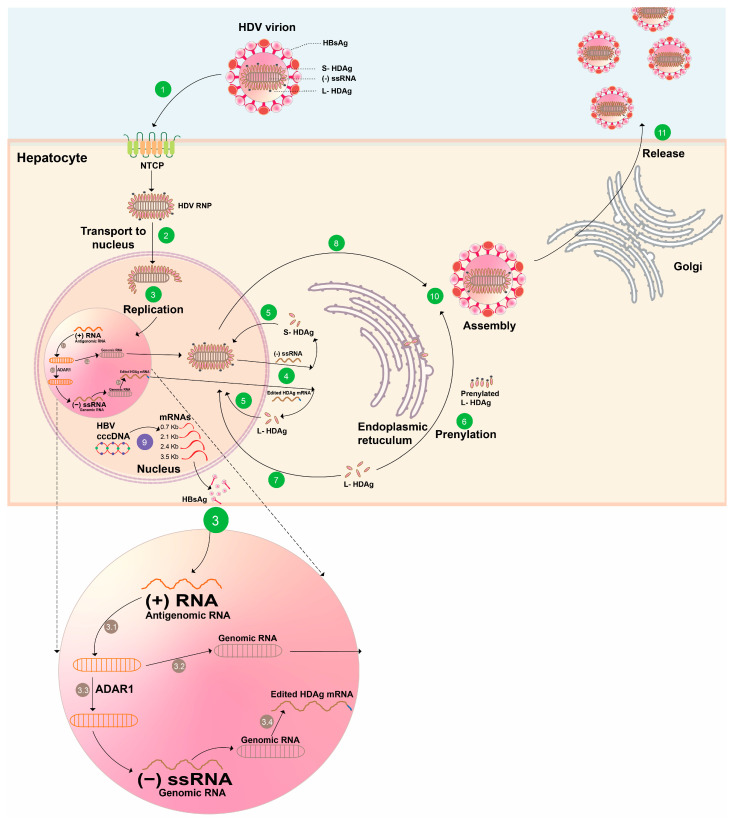
Hepatitis D viral life cycle. The figure represents a cell infected with HBV and HDV. HDV virions are enveloped by the hepatitis B virus (HBV) HBsAg, which can attach to hepatocytes and bind to sodium taurocholate co-transporting polypeptide (NTCP) to allow endocytosis. Then, the HDV ribonucleoprotein (RNP) complex, which is composed of circular HDV RNA, as well as the large (L) and small (S) hepatitis delta antigen (HDAg), is released into the cytoplasm (1), translocated to the nucleus by HDAg (2), where HDV replication and transcription occur (3). Transcription is dependent on host polymerases. The HDV genomic RNA serves as a template to produce antigenomic RNA via RNA polymerase II. (3.1) The antigenomic RNA is then used by RNA polymerase II to produce new genomic RNAs. (3.2) The antigenomic RNA is also modified by the ADAR1 enzyme, which leads to the elimination of the stop codon of the S-HDAg. (3.3) The modified antigenomic RNA is replicated into the genomic RNA, thus inducing the transcription of the modified HDAg mRNA (3.4), which is exported to the endoplasmic reticulum (ER) (4), where it is translated into S-HDAg (5) and L-HDAg. The L-HDAg requires prenylation before assembly into the RNP complex and is translocated to the nucleus (6). S-HDAg supports HDV replication and is recycled into the nucleus (7). New RNPs via new HDAg molecules are formed and exported into the cytoplasm (8). New HDV RNP is associated with HBV subviral particles (SVPs), produced from HBV mRNA (9), enriched in L-HBsAg compared to classical sphere-SVPs (10), and are secreted through the ESCRT/MVB pathway like infectious viral particles (11) [6,7].

Hepatitis D virus (HDV) is a satellite virus that requires the presence of a helper virus, notably the hepatitis B virus (HBV), for its replication, exclusively for enveloping its virion and for infecting susceptible cells [1,2]. It is a very peculiar virus that shares many characteristics with plant viroids, which are small, circularized, non-coding RNAs able to replicate in plants (Figure 1) [3].

Infection with HDV is frequently associated with a severe disease, which may present with fulminant hepatitis or a more rapid progression to cirrhosis and hepatocellular carcinoma (HCC), when compared to HBV mono-infection [4,5].

In addition to the frequent lack of diagnosis of HDV infection in patients at risk, its viral characteristics have led to HDV being considered as a neglected virus. This review aims to identify the most important gaps in knowledge and distinctive features in the study of this enigmatic virus.

## 2. HDV and the Kolmioviridae Family

### 2.1. Other Deltavirus-like Agents

Since its discovery in 1977 [8], HDV was, until recently, thought to be an isolated member of the *Deltavirus* genus. The discovery of HDV-like agents in birds in 2018, and then in snakes and other reptiles, as well as HDV-like sequences in other mammals and even in termites, led to the inclusion of this group of viruses in the family *Kolmioviridae* [1,9,10]. The RNA genome of HDV is single-stranded, of negative polarity, circularized, paired (because of high self-complementarity) and consists of around 1700 nt, and thus is the smallest known virus able to infect animal cells.

These HDV viruses share several characteristics (Table 1):

- Ribozyme activity, shared also with plant viroids, both in their genomic and antigenomic RNA [11].
- A genome that encodes a single protein (in contrast to viroids), which exists in two forms: the small (S) and large (L) hepatitis delta antigen (HDAg) that differ by 19–20 additional amino acids at the C-terminal end. The L-HDAg is produced by the cellular edition of a stop codon of the viral RNA to encode a tryptophan [10]. Other members, except the rodent *Deltavirus*, express an antigen similar to the one found in HDV.
- The circularized quasi double-stranded (by base pairing) RNA is processed as double-stranded DNA by the host RNA polymerases in the nucleus to produce mRNA and genomic and antigenomic RNA.
- The only dependence of some of the members of the *Kolmioviridae* family on another virus is their use of the envelope protein produced by the helper virus. In the case of HDV, this envelope is provided by the subviral particles produced in excess by HBV [12,13]. This allows HDV to enter the hepatocyte via the sodium-taurocholate co-transporting polypeptide (NTCP) receptor (Figure 1) [10].

### 2.2. HDV Replication

Several aspects in the replication of HDV (Figure 1) are still poorly understood [7].

Firstly, the trafficking of the HDV virion/genome into the cell and its nucleus remains a highly debated issue. It has been shown that the L-HDAg possesses a clathrin interaction motif, and this interaction may be involved in the viral endocytosis and exocytosis [14].

A second major unresolved issue concerns how a quasi-double stranded RNA genome is transcribed by the host RNA polymerases. The redirecting of host RNA polymerases to transcribe RNA is a unique ability of viroid and viroid-like RNAs, probably by forming a modified enzymatic complex [15].

Regarding the regulation of the rolling/circle replication, multiple lines of evidence suggest that the Polymerase II participates in HDV replication, although the two other RNA polymerases probably also participate. In addition, the HDV ribozyme activity and a host RNA ligase, or a self-ligase activity of the HDV RNA, are also needed [15].

Finally, the molecular mechanism by which host RNA polymerases discriminate between genomic replication and mRNA synthesis is poorly defined [15,16].

Although several studies have been conducted on this viral life cycle, many gaps of knowledge remain for this enigmatic virus.

### 2.3. HDV Origin

The origin of the members of the *Kolmioviridae* family may be related to that of viroids, since they share many characteristics. Due to the small size of their genome, it has been proposed that they are derived from the RNA world that likely preceded the cellular world [3]. However, the wide distribution of delta-like agents in different taxa with great divergence suggests that these members may be derived from the cellular transcriptome [17].

With respect to HDV, most of the known HDV genotypes are found in Africa, which may suggest an Old-World origin for this virus [9]. However, the most divergent HDV genotype is the American HDV-3. This may also be due to an independent introduction or a rapid co-divergence, as for the HBV American genotypes [18].

### 2.4. Cellular Edition of HDV RNA: Converting an Enemy into a Friend?

As stated before, HDV encodes the S-HDAg and, upon editing of its antigenome RNA, a stop codon is abrogated to encode 19–20 additional residues, which are quite variable among different genotypes [19]. The antigenome RNA is edited by a specific host enzyme, with the adenosine deaminase acting on RNA (ADAR), specifically ADAR-1 [20]. Although ADAR-1 is supposed to exert an antiviral effect for other viruses, site-specific editing at the amber UAG stop codon (W-site) has a proviral effect, promoting the production of the L-HDAg. The extra amino acids encoded by this edition are the ones that interact with specific amino acids of the HBV surface antigen (HBsAg). This enzyme is thus indispensable for HDV replication. However, its overexpression inhibits HDV replication [21]. The ADAR-1 editing activity has been shown to be finely tuned by HDV [22], suggesting a complex feedback regulation.

Upon analysis of plasma samples from patients infected with different HDV genotypes, different editing capacities were found depending on the viral genotype [20]. Further studies are needed to assess if the levels of HDV RNA edition and the L-HDAg produced might be related to the differential pathogenicity of HDV genotypes.

### 2.5. HDV Genotypes and Pathogenicity

Eight HDV genotypes have been described, and for some of them, subgenotypes have also been reported. The genotypes 4–8 (HDV-4 to 8) were described in 2017 [23]. Recombinant viruses (between HDV-1 and HDV-2) have also been described [24]. The intergenotypic divergence is more than 20% [25]. Multiple lines of evidence suggest that any of the 10 HBV genotypes, in addition to other mammalian hepadnaviruses, such as woodchuck hepatitis virus (WHV), can support the replication of any of the HDV genotypes [26]. However, natural infection of the woodchuck with HDV has not been documented.

HDV-3 is the most divergent of the HDV genotypes and is endemic in South America, particularly in the Amazon Basin. Interestingly, the American HBV genotype F, frequently found as the co-infecting virus, is also the most divergent of the human HBV genotypes. Infection with HDV-3 has been frequently associated with episodes of fulminant hepatitis [27]. It is also accepted that infection with HDV-1 or 3 is more aggressive than with HDV-2 and 4 [25]. In another study, patients infected with HDV-5 were found to be more prone to develop cirrhosis [28]. However, the existence of a differential pathogenicity according to the HDV-infecting genotypes and the degree of pathogenicity of HDV-6-8 is still unknown. A lower number of complete genome sequences is available for some of the newer genotypes: a search in GenBank for HDV complete genome sequences, for example, showed more than 700 sequences for HDV-1 and 90 for HDV-2, while less than 30 sequences were available for HDV-8. More studies are needed to assess if there is a differential pathogenicity depending on the HDV genotype.

### 2.6. HDV Envelope Provided by Other Viruses?

It is known that HDV utilizes the HBV envelope protein to envelope its own genome. Perez-Jimenez et al. (2019) showed that in vitro, envelope glycoproteins from *vesiculovirus*, *flavivirus*, and *hepacivirus* were able to package the HDV ribonucleoprotein (RNP), with subsequent egress from the cell and entry into permissive ones [29]. Moreover, the liver of HCV-infected humanized mice was able to support HDV infection for several months. These experimental results prompted the question of whether HCV infection in humans was able to support HDV dissemination in vivo.

Chemin et al. (2021) analyzed a cohort of 160 HCV-infected patients without any serological markers of HBV infection. Antibodies against HDV were found in two patients, and HDV RNA (genotype 1) was found in one [30]. Two other studies found the presence of anti-HDV antibodies in: (1) blood donors with HCV markers and (2) in a patient co-infected with HCV and HIV, without evidence of HBV co-infection. No HDV RNA was detected in any of the samples [31,32]. Another study found 8/316 samples positive for anti-HDV antibodies in HCV-infected patients without HBsAg. The eight samples were, however, positive for antibodies against the HBV core protein [33], thus not excluding the presence of a seropositive occult HBV infection. Indeed, HBV occult infection, i.e., the presence of intrahepatic replication-competent HBV DNA in individuals testing negative for serum HBsAg [34,35], was not investigated in any of these studies. Occult HBV infection is even more frequent in patients also infected with HCV [36]; therefore, the presence of HBV antigens in the liver, even at very low levels, could not be completely excluded.

Sjögren’s disease (SjD) is a chronic autoimmune disease characterized by decreased tear and/or saliva production. HDV genome and antigens have been found in salivary glands of patients with SjD in two independent studies, in the absence of any HBV marker. However, HCV infection was not detected in some of these patients, not excluding the possibility that this virus might be co-infecting those patients [37,38]. A member of the *Kolmioviridae* family, the snake *Deltavirus*, has been shown to use the envelope proteins at least from *reptarenavirus* and *hartmaniviruses* for its replication [39]. In contrast, the rodent *Deltavirus* does not seem to encode any antigen and might not need a helper virus for its propagation [40]. However, more recent studies show the interaction of the rodent *Deltavirus* with the envelope of vesicular stomatitis virus and herpesvirus [41].

All these pieces of evidence suggest that the members of the *Kolmioviridae* family are composed mainly, if not all, of satellite viruses. The main function of the helper virus is to provide an envelope for its RNPs. For some of these viruses, different helper viruses may provide it. In some other cases, the presence of this virus might be dispensable. In the specific case of HDV, the evidence suggesting viruses other than HBV as helper for co-infection is emerging but not conclusive. For the case of HCV co-infection with HDV, in the apparent absence of HBV co-infection, an occult HBV infection could not be discarded in those studies so far. However, the reports of the presence of anti-HDV antibodies in HCV-infected patients warrant further studies in such populations, particularly in regions with high HBV/HDV endemicity, to assess if the HCV-infected population might also be an unexplored source of HDV infection in some settings.

## 3. HDV Epidemiology

### What Is the Real Prevalence of HDV Infection?

A recent systematic review assessed the number of HDV-infected/exposed persons worldwide to be 12 million [42]. A report published the same year estimated this number to be 48–60 million [43], with a “revisited” estimation of 50 million the same year [44]. An adjusted estimate, by analyzing more precisely 25 countries, showed a high degree of uncertainty in HDV prevalence and suggested even lower numbers than 12 million [45]. These studies show the many gaps in knowledge of the real prevalence of HDV infection/exposure [46]. In addition, a sharp decline in HDV prevalence is expected because of HBV vaccination, as observed for the helper virus [47]. Epidemiological data in many countries, however, remain scant. For example, in Mongolia, a global hotspot for HDV, reported anti-HDV seroprevalence among HBsAg carriers is roughly 60% [48]. This strikingly high burden underscores how the global estimate of 12 million may underestimate the true number in endemic regions, highlighting the urgent need for more comprehensive, geographically diverse surveillance. Risk factors for HDV infection are also not completely known. Surveillance data at the country level, public health testing approaches (including reflex testing of HBV-positive cases), and widely available treatment options are warranted for a better control of HDV infection [49]. It has been shown that reflex testing (testing for HDV infection in each HBsAg positive patient) improves HDV diagnostics at an earlier stage, reducing disease development and thus the costs of clinical management of the patients [49,50]. On the other hand, many prevalence studies on HDV are limited to detecting HDV antibodies: these studies overestimate the real prevalence of active HDV infection.

## 4. Clinical Presentation

### 4.1. Superinfection for HDV Persistence: Importance of (Innate) Immune Response

Not all HDV co-infections lead to viral persistence. It is generally accepted that simultaneous infection with HBV and HDV mostly leads to the eradication of both viruses. In contrast, superinfection with HDV in a patient with chronic HBV infection is more prone to develop a chronic HDV infection [51]. HBsAg (large antigen including the receptor-binding preS1 region) produced exclusively by integrated HBV DNA can effectively support dissemination of HDV. Indeed, HDV replication seems highly dependent on HBV integration [25,52,53]. This may explain why simultaneous infection with both viruses does not lead to a chronic HDV infection. Since the majority of HBV infection in adulthood does not establish a chronic infection, preventing the dissemination of HDV, clearance of the HBV-infected hepatocytes may also lead to HDV clearance. However, this may not apply to co-infection acquired during childhood and specifically the neonatal period, when the probability of a chronic HBV infection is around 90% without prophylactic measures [47]. A study on vertical transmission of HBV and HDV showed a low frequency of HDV vertical transmission [54]. However, information is too scarce to address this important point [55,56].

However, one could speculate that in a simultaneous co-infection, HBV could be cleared, but HDV infection could be maintained as a mono-infection in the hepatocyte, without viral dissemination to other cells. HDV can persist in hepatocytes before subsequent infection with HBV [57]. HDV mono-infection has been observed in hepatic cells in patients with chronic HBV/HDV co-infection [58]. Similar results have been recently reported by the use of spatial transcriptomics, in which HBV+ and HDV+ cells were mutually exclusive [59]. An intact innate immune response in the host may play a role in clearing HDV infection [6]. This response may be altered in chronic HBV infection, favoring HDV persistence in the case of superinfection [8]. Indeed, hepatocytes from HDV/HBV-infected patients present an increased expression of genes such as CD40 and SRC Proto-Oncogene (SRC), which have been previously reported to have an inhibitory role in HBV replication, as well as the downregulation of anti-inflammatory genes including RAR related orphan receptor A (RORA), hepatocyte growth factor (HGF) and LYN Proto-Oncogene (LYN) [60]. HDV infection inhibits HBV replication by inducing a strong type-I IFN response, in turn inducing the IFN stimulating genes RSAD2 (Viperin) and IF178 (MxA) [61]. However, HDV interference with HBV can occur by both IFN-dependent and independent mechanisms [62].

### 4.2. Viral Biomarkers: Atypical Presentation

Biomarkers of HDV involve the detection and quantification of viral RNA and antigens reflecting viral replication and infection status. The main biomarkers include HDV RNA (synthesized during viral replication), HDAg (produced from the HDV genome), and anti-HDV antibodies (reflecting host immune response). These biomarkers are essential for diagnosis, evaluating disease progression, and monitoring antiviral therapy efficacy [63,64].

During HDV co- or superinfection, HBV DNA levels are typically suppressed due to HDV’s inhibitory effect on HBV replication. However, HBsAg levels often remain stable or are only slightly reduced, as HDV requires HBsAg for its assembly and release and HBsAg production is mostly sustained by HBV integration in co-infected patients. This dissociation—low HBV DNA but persistent HBsAg—reflects the dominance of HDV replication over HBV and complicates antiviral therapy aimed solely at HBV suppression. Monitoring biomarkers for both viruses is therefore crucial for assessing infection dynamics and treatment response [65]. Emerging HBV biomarkers, serum HBV RNA and HBV core related antigen (HBcrAg), have been found to correlate with the intrahepatic covalently closed circular DNA levels and transcriptional activity in chronic HBV monoinfection [66]. In CHD patients negative for Hepatitis B e antigen (HBeAg), a divergent pattern was observed: 68% had undetectable serum HBV RNA and positive HBcrAg quantification [67]. These results stress the importance of assessing the suitability of HBV biomarkers in the setting of HDV co-infection.

### 4.3. Disease Progression: Oncogenic Mechanisms Distinct from HBV Ones

HDV co-infection increases the risk of cirrhosis, HCC, hepatic decompensation, mortality, and the necessity for liver transplant [61,68,69]. A systematic review showed that patients with active HDV infection (HDV RNA-positive) are at greater risk for liver disease than those without active infection [70]. Although some limitations apply to this study, it is clear that HDV co-infection increases the risk of liver disease when compared to HBV mono-infection [71]. In cirrhotic HBV-infected patients, HDV also increases the risk of developing HCC [72]. Another study showed that in HBV-infected patients treated with nucleos(t)ide analogues (NUCs), one of the factors associated with HCC development, in addition to cirrhosis, was the presence of HDV RNA positivity [73]. It is estimated that HDV infection increases the risk of HCC progression threefold [74]. Moreover, it has been shown that HDV infection increases the risk of HCC, independently of the presence of cirrhosis [61]. This supports the concept of pro-oncogenic factors related to HDV infection [25]. Transcriptomic analyses have shown the up-regulation of genes involved in genome instability or the silencing of oncosuppressor genes in HDV-related tumoral liver tissues [25].

Many of the mechanisms underlying HCC development by HDV infection are still unknown. Several studies suggest that HDAg may play a role in hepatocyte transformation. L-HDAg appears to activate signal transducer and activator of transcription (STAT)-3 or nuclear factor kappa B (NF-κB) induced by oxidative stress, inducing stress of the endoplasmic reticulum, necrotizing inflammation, and an increase in reactive oxygen species production. All these factors may lead to HCC development [25,74]. A recent study has shown that several cellular protein kinases were dysregulated by the expression of both forms of HDAg, some of these targets have been associated with HCC development [75].

A comparative analysis of hepatitis virus-associated HCC showed that the molecular signature of HDV-HCC is distinct from HBV-HCC. The dysregulation of genes associated with DNA damage and repair suggests that genetic instability may be a key mechanism of HDV-induced carcinogenesis [76]. Transcriptomic analyses indicate that HDV-HCC displays a marked enrichment in immunological signaling pathways relative to other HCC etiologies, potentially contributing to the unique biological and clinical phenotype characteristic of HDV-HCC [77]. A microarray study on HDV-related HCC identified 7 upregulated genes, related to tumorigenesis, in agreement with the evidence suggesting a molecular pathway to HCC development, independent of HBV [78]. A bioinformatic analysis identified many upregulated genes: in particular, two upregulated genes and three downregulated ones were associated with worse progression to HCC [79]. HDV has been recently classified as carcinogenic to humans by the International Agency of Research on Cancer and World Health Organization [80].

## 5. Prevention and Treatment

Considering that HDV depends on HBsAg for its viral cycle, currently available HBV vaccines can indirectly prevent HDV infection by preventing HBV. However, this does not confer protection against HDV superinfection in HBV-infected individuals. In this context, it has been reported that in small animal models, the use of a vaccine containing antigens from HBsAg and HDAg was able to induce antibodies that could protect against HBV/HDV co-infection or HDV superinfection [81]. Thus, similar studies could accelerate the development of an HDV vaccine, which remains an unmet medical need. Few advances have occurred in the field of a therapeutic vaccine against HDV. In contrast, several studies have been performed on the development of therapeutic vaccines against HBV, although a limited number have progressed to clinical trials, still without success in achieving functional cure [82].

As chronic hepatitis D (CHD) is the most aggressive form of viral hepatitis, antiviral therapy aims to decrease the risk of disease progression (i.e., cirrhosis, decompensation, HCC) by reducing HDV replication and inflammation while managing HBV activity. Although NUC therapy does not suppress HDV replication, it is recommended in patients with decompensated cirrhosis, compensated cirrhosis with detectable HBV DNA, and patients without cirrhosis if HBV DNA levels are higher than 2000 IU/mL [83].

Although not formally approved, Pegylated interferon-α (peg-IFN-α) was, until recently, the only therapeutic option for CHD. The European Association for the Study of the Liver (EASL) recommends peg-IFN-α for 48 weeks as the preferred schedule, with personalized treatment duration based on HDV RNA and HBsAg kinetics, as well as treatment tolerability [83].

Bulevirtide (BLV), a myristoylated lipopeptide derived from 47 amino acids of the preS1 domain of HBsAg that blocks viral entry, was approved in 2020 by the European Medicines Agency (EMA) as CHD therapy [84]. Although the optimal duration and dose of BLV monotherapy treatment remain to be determined, it can be employed until a clinical benefit is observed. It has been shown that a 3-year course of BLV may cure HDV infection in cirrhotic patients [85]. The cases of sustained HDV clearance can occur with persistence of HBsAg [85,86]. Moreover, the combination of BLV and peg-IFN-α is an option in patients without intolerance or contraindications, as it has shown to be associated with a higher HDV RNA decline and higher sustained virologic response after treatment cessation as compared to BLV monotherapy [87]. Nonetheless, given the practical constraint that BLV needs to be administered by daily subcutaneous injection, the evaluation of new small-molecule NTCP inhibitors that could be taken orally is an active field of research [88].

In addition to the above-described standard of care, other approaches are currently being evaluated and have shown promising results during clinical evaluation. Some of these include the monoclonal antibodies against HBsAg BJT-778 and tobevibart, the latter one employed in combination with the siRNA against HBV transcripts, elibsiran [83,89,90]. A recent study shows that REP 2139-Mg, a nucleic acid polymer that can block HBV subviral particle assembly and HDV replication, was safe and effective in HDV patients with advanced chronic liver disease, achieving a sustained HDV virological response and HBV functional cure in a subset of patients [91]. The hope is that the study of drugs with different mechanisms of action could guide the design of the most appropriate combinations.

## 6. Concluding Remarks

Despite significant advances in the study of HDV, substantial enigmas and knowledge gaps persist, hindering a complete understanding and effective control of this disease. The origin and evolution of HDV remain unclear, with its relationship to other deltavirus-like agents and viroids still not fully understood. The virus exhibits a unique reliance on HBV, although potential interactions with other helper viruses add further enigma to the puzzle, while evidence for alternative envelopes in vivo still remains inconclusive. Epidemiologically, its global prevalence is still poorly defined due to inadequate surveillance and testing. The mechanisms underlying HDV-induced hepatocellular carcinoma, its variable pathogenicity among genotypes, and the role of host factors in disease progression are still incompletely characterized. These unresolved questions, compounded by the role of host factors like the ADAR-1 editing enzyme and the immune response in viral persistence, directly challenge the development of a specific vaccine and optimal curative strategies. Furthermore, the development of effective therapeutic and preventive strategies is hindered by these scientific gaps, underlining the need for continued research to unravel the mysteries of HDV infection. Overcoming these knowledge gaps will not only clear the final enigmas of this fascinating virus but also open the door to comprehensive curative strategies for one of the most aggressive forms of hepatitis.

## Figures and Tables

**Table 1:** HDV enigmas and gaps.

Characteristics	Open Questions
Other deltaviruses	The emerging family *Kolmioviridae*
2. HDV replication	Host polymerases’ involvement, regulation of rolling circle replication
3. Origin	Derived from the cellular transcriptome? An African origin for the human HDV?
4. RNA editing by ADAR	Converting an enemy into a friend?
5. Genotypes and pathogenicity	Why is genotype 3 particularly pathogenic?
6. Ability to infect hepatocyte without HBV	Efficiency of HDV dissemination independently of HBV? A role for HCV in HDV dissemination?
7. Real worldwide HDV prevalence	More studies and testing needed. Hotspots of prevalence?
8. Superinfection vs. coinfection	Why is co-infection less likely? Influence of (innate) immunity?
9. Viral biomarkers	Tailor-made for HBV/HDV co-infection?
10. HCC development	Oncogenic mechanisms distinct from HBV ones
11. Prevention and treatment	HDV vaccine? Direct targeting antivirals? Bulevirtide non-effective for HBV, but very promising for HDV

## Data Availability

No new data were created or analyzed in this study.

## Abbreviations

The following abbreviations are used in this manuscript:

ADAR	Adenosine deaminase acting on RNA
BLV	Bulevirtide
CHD	Chronic hepatitis D
EASL	European Association for the Study of the Liver
EMA	European Medicines Agency
HBV	Hepatitis B virus
HBcrAg	Hepatitis B core related antigen
HBeAg	Hepatitis B e antigen
HBsAg	Hepatitis B surface antigen
HCC	Hepatocellular carcinoma
HCV	Hepatitis C virus
HDAg	Hepatitis D antigen
L-HDAg	Hepatitis D large antigen
HDV	Hepatitis D virus
HGF	hepatocyte growth factor
NTCP	Sodium taurocholate co-transporting polypeptide
NF-κB	Nuclear factor kappa B
NUC	Nucleos(t)ide analogues
peg-IFN-α	Pegylated interferon-α
RNP	Ribonucleoprotein
RORA	RAR related orphan receptor A
S-HDAg	Hepatitis D small antigen
SjD	Sjögren’s disease
SRC	SRC Proto-Oncogene
SVPs	Subviral particles

## References

[1] Perez-Vargas J., Pereira de Oliveira R., Jacquet S., Pontier D., Cosset F.L., Freitas N. (2021). HDV-Like Viruses. Viruses.

[2] Sureau C., Guerra B., Lanford R.E. (1993). Role of the large hepatitis B virus envelope protein in infectivity of the hepatitis delta virion. J. Virol..

[3] Flores R., Navarro B., Serra P., Di Serio F. (2022). A scenario for the emergence of protoviroids in the RNA world and for their further evolution into viroids and viroid-like RNAs by modular recombinations and mutations. Virus Evol..

[4] Negro F., Lok A.S. (2023). Hepatitis D: A Review. JAMA.

[5] Roca Suarez A.A., Batbold E., Bartosch B., Dashdorj N., Testoni B., Zoulim F. (2023). Emerging anti-HDV drugs and HBV cure strategies with anti-HDV activity. Liver Int..

[6] Jiang B., Himmelsbach K., Ren H., Boller K., Hildt E. (2015). Subviral Hepatitis B Virus Filaments, like Infectious Viral Particles, Are Released via Multivesicular Bodies. J. Virol..

[7] Zi J., Gao X., Du J., Xu H., Niu J., Chi X. (2022). Multiple Regions Drive Hepatitis Delta Virus Proliferation and Are Therapeutic Targets. Front. Microbiol..

[8] Rizzetto M., Canese M.G., Arico S., Crivelli O., Trepo C., Bonino F., Verme G. (1977). Immunofluorescence Detection of New Antigen-Antibody System (Delta/Anti-Delta) Associated to Hepatitis-B Virus in Liver and in Serum of Hbsag Carriers. Gut.

[9] Bergner L.M., Orton R.J., Broos A., Tello C., Becker D.J., Carrera J.E., Patel A.H., Biek R., Streicker D.G. (2021). Diversification of mammalian deltaviruses by host shifting. Proc. Natl. Acad. Sci. USA.

[10] Khalfi P., Denis Z., McKellar J., Merolla G., Chavey C., Ursic-Bedoya J., Soppa L., Szirovicza L., Hetzel U., Dufourt J. (2024). Comparative analysis of human, rodent and snake deltavirus. replication. PLoS Pathog..

[11] De la Peña M., Gago-Zachert S. (2022). A life of research on circular RNAs and ribozymes: Towards the origin of viroids, deltaviruses and life. Virus Res..

[12] Rizzetto M., Hoyer B., Canese M.G., Shih J.W., Purcell R.H., Gerin J.L. (1980). Delta Agent: Association of delta antigen with hepatitis B surface antigen and RNA in serum of delta-infected chimpanzees. Proc. Natl. Acad. Sci. USA.

[13] Sureau C., Lanford R. (1993). Analysis of hepatitis B virus envelope proteins in assembly and infectivity of human hepatitis delta virus. Prog. Clin. Biol. Res..

[14] Chiou W.C., Lu H.F., Chen J.C., Lai Y.H., Chang M.F., Huang Y.L., Tien N., Huang C. (2022). Identification of a novel interaction site between the large hepatitis delta antigen and clathrin that regulates the assembly of genotype III hepatitis delta virus. Virol. J..

[15] Kalantidis K., Tselika M., Kallemi P., Bardani E., Kryovrysanaki N., Katsarou K. (2025). Derailing the host machinery to achieve replication: How viroid and viroid-like RNAs successfully copy their genomes in hostile territory. RNA Biol..

[16] Whelan M., Pelchat M. (2022). Role of RNA Polymerase II Promoter-Proximal Pausing in Viral Transcription. Viruses.

[17] Netter H.J., Barrios M.H., Littlejohn M., Yuen L.K.W. (2021). Hepatitis Delta Virus (HDV) and Delta-Like Agents: Insights Into Their Origin. Front. Microbiol..

[18] Paraskevis D., Angelis K., Magiorkinis G., Kostaki E., Ho S.Y., Hatzakis A. (2015). Dating the origin of hepatitis B virus reveals higher substitution rate and adaptation on the branch leading to F/H genotypes. Mol. Phylogenet. Evol..

[19] Guo H., Li Q., Li C., Hou Y., Ding Y., Liu D., Ni Y., Tang R., Zheng K., Urban S. (2023). Molecular determinants within the C-termini of L-HDAg that regulate hepatitis D virus replication and assembly. JHEP Rep..

[20] Dziri S., Rodriguez C., Gerber A., Brichler S., Alloui C., Roulot D., Dény P., Pawlotsky J.M., Gordien E., Le Gal F. (2021). Variable In Vivo Hepatitis D Virus (HDV) RNA Editing Rates According to the HDV Genotype. Viruses.

[21] Datta R., Adamska J.Z., Bhate A., Li J.B. (2023). A-to-I RNA editing by ADAR and its therapeutic applications: From viral infections to cancer immunotherapy. Wiley Interdiscip. Rev. RNA.

[22] Casey J.L. (2012). Control of ADAR1 editing of hepatitis delta virus RNAs. Curr. Top. Microbiol. Immunol..

[23] Le Gal F., Brichler S., Drugan T., Alloui C., Roulot D., Pawlotsky J.M., Dény P., Gordien E. (2017). Genetic diversity and worldwide distribution of the deltavirus genus: A study of 2152 clinical strains. Hepatology.

[24] Chowdhury S., Jacobsen C., Depledge D.P., Wedemeyer H., Sandmann L., Kefalakes H. (2025). Sequence analysis of the hepatitis D virus across genotypes reveals highly conserved regions amidst evidence of recombination. Virus Evol..

[25] Salpini R., D’Anna S., Piermatteo L., Svicher V. (2022). Novel concepts on mechanisms underlying Hepatitis Delta virus persistence and related pathogenesis. J. Viral Hepat..

[26] Beghin J., Meier-Stephenson V. (2023). Does hepatitis delta virus have a preference for hepatitis B virus genotype? A systematic review of the literature. J. Viral Hepat..

[27] Paraná R., Pujol F.H. (2019). Clinical and Virological Heterogeneity of Hepatitis Delta in the Amazonia: More Questions Than Answers. Clin. Liver Dis..

[28] Roulot D., Brichler S., Layese R., BenAbdesselam Z., Zoulim F., Thibault V., Scholtes C., Roche B., Castelnau C., Poynard T. (2020). Deltavir study group. Origin, HDV genotype and persistent viremia determine outcome and treatment response in patients with chronic hepatitis delta. J. Hepatol..

[29] Perez-Vargas J., Amirache F., Boson B., Mialon C., Freitas N., Sureau C., Fusil F., Cosset F.-L. (2019). Enveloped viruses distinct from HBV induce dissemination of hepatitis D virus in vivo. Nat. Commun..

[30] Chemin I., Pujol F.H., Scholtès C., Loureiro C.L., Amirache F., Levrero M., Zoulim F., Pérez-Vargas J., Cosset F. (2021). Preliminary Evidence for Hepatitis Delta Virus Exposure in Patients Who Are Apparently Not Infected with Hepatitis B Virus. Hepatology.

[31] Cappy P., Lucas Q., Kankarafou N., Sureau C., Laperche S. (2021). No Evidence of Hepatitis C Virus (HCV)-Assisted Hepatitis D Virus Propagation in a Large Cohort of HCV-Positive Blood Donors. J. Infect. Dis..

[32] Crobu M.G., Ravanini P., Impaloni C., Martello C., Bargiacchi O., Di Domenico C., Faolotto G., Macaluso P., Mercandino A., Riggi M. (2024). Hepatitis C Virus as a Possible Helper Virus in Human Hepatitis Delta Virus Infection. Viruses.

[33] Pflüger L.S., Schulze Zur Wiesch J., Polywka S., Lütgehetmann M. (2021). Hepatitis delta virus propagation enabled by hepatitis C virus-Scientifically intriguing, but is it relevant to clinical practice?. J. Viral Hepat..

[34] Mariscal L.F., Rodríguez-Iñigo E., Bartolomé J., Castillo I., Ortiz-Movilla N., Navacerrada C., Pardo M., Pérez-Mota A., Graus J., Carreño V. (2004). Hepatitis B infection of the liver in chronic hepatitis C without detectable hepatitis B virus DNA in serum. J. Med. Virol..

[35] Saitta C., Pollicino T., Raimondo G. (2022). Occult Hepatitis B Virus Infection: An Update. Viruses.

[36] Pan Y., Jia Z., Zhang Y., Wu Y., Jiang J. (2025). Estimates of the global prevalence of occult hepatitis B virus infection in population under 18 years old: A systematic review and meta-analysis. Hepatol. Int..

[37] Weller M.L., Gardener M.R., Bogus Z.C., Smith M.A., Astorri E., Michael D.G., Michael D.A., Zheng C., Burbelo P.D., Lai Z. (2016). Hepatitis Delta Virus Detected in Salivary Glands of Sjögren’s Syndrome Patients and Recapitulates a Sjögren’s Syndrome-Like Phenotype in Vivo. Pathog. Immun..

[38] Hesterman M.C., Furrer S.V., Fallon B.S., Weller M.L. (2023). Analysis of Hepatitis D Virus in Minor Salivary Gland of Sjögren’s Disease. J. Den. Res..

[39] Szirovicza L., Hetzel U., Kipar A., Martinez-Sobrido L., Vapalahti O., Hepojoki J. (2020). Snake Deltavirus Utilizes Envelope Proteins of Different Viruses to Generate Infectious Particles. mBio.

[40] Paraskevopoulou S., Pirzer F., Goldmann N., Schmid J., Corman V.M., Gottula L.T., Schroeder S., Rasche A., Muth D., Drexler J.F. (2020). Mammalian deltavirus without hepadnavirus coinfection in the neotropical rodent *Proechimys semispinosus*. Proc. Natl. Acad. Sci. USA.

[41] McKellar J., Fouillen A., Lyonnais S., Seigneuret F., Blanchard M.P., Trullo A., Kumarasinghe L., De Rossi S., Sleiman R., Desagher S. (2025). Deltaviruses spread through a viral Trojan Horse. bioRxiv.

[42] Stockdale A.J., Kreuels B., Henrion M.Y.R., Giorgi E., Kyomuhangi I., de Martel C., Hutin Y., Geretti A.M. (2020). The global prevalence of hepatitis D virus infection: Systematic review and meta-analysis. J. Hepatol..

[43] Miao Z., Zhang S., Ou X., Li S., Ma Z., Wang W., Peppelenbosch M.P., Liu J., Pan Q. (2020). Estimating the Global Prevalence, Disease Progression, and Clinical Outcome of Hepatitis Delta Virus Infection. J. Infect. Dis..

[44] Miao Z., Pan Q. (2020). Revisiting the estimation of hepatitis D global prevalence. J. Hepatol..

[45] Polaris Observatory Collaborators (2024). Adjusted estimate of the prevalence of hepatitis delta virus in 25 countries and territories. J. Hepatol..

[46] Hayashi T., Takeshita Y., Hutin Y.J., Harmanci H., Easterbrook P., Hess S., van Holten J., Oru E.O., Kaneko S., Yurdaydin C. (2021). The global hepatitis delta virus (HDV) epidemic: What gaps to address in order to mount a public health response?. Arch. Public Health.

[47] Pujol F.H., Toyé R.M., Loureiro C.L., Jaspe R.C., Chemin I. (2023). Hepatitis B eradication: Vaccine as a key player. Am. J. Transl. Res..

[48] Chen X., Oidovsambuu O., Liu P., Grosely R., Elazar M., Winn V.D., Fram B., Boa Z., Dai H., Dashtseren B. (2017). A novel quantitative microarray antibody capture assay identifies an extremely high hepatitis delta virus prevalence among hepatitis B virus-infected mongolians. Hepatology.

[49] Fuentes A., Estévez-Escobar M., De Salazar A., Escolano E.R., Montiel N., Macías M., Alados J.C., Aguilar J.C., Pérez A.B., Baena P.B. (2025). Double reflex testing improves the efficacy and cost effectiveness of hepatitis delta diagnosis in southern Spain. Sci. Rep..

[50] Wong R.J., Gish R.G., Jacobson I.M., Lim J.K., Rock M., Kinyik-Merena C., Ma H., Smith N., Kim C. (2026). Impact of Double Reflex Testing and Linkage to Treatment on Clinical Outcomes of Chronic Hepatitis Delta Virus Infection in the United States. J. Viral Hepat..

[51] Negro F. (2014). Hepatitis D virus coinfection and superinfection. Cold Spring Harb. Perspect. Med..

[52] Ringlander J., Strömberg L.G., Stenbäck J.B., Andersson M.E., Abrahamsson S., Skoglund C., Castedal M., Larsson S.B., Rydell G.E., Lindh M. (2024). Enrichment Reveals Extensive Integration of Hepatitis B Virus DNA in Hepatitis Delta Virus-Infected Patients. J. Infect. Dis..

[53] Zoulim F., Chen P.J., Dandri M., Kennedy P.T., Seeger C. (2024). Hepatitis B virus DNA integration: Implications for diagnostics, therapy, and outcome. J. Hepatol..

[54] Sellier P.O., Maylin S., Brichler S., Berçot B., Lopes A., Chopin D., Pogliaghi M., Munier A.L., Delcey V., Simoneau G. (2018). Hepatitis B Virus-Hepatitis D Virus mother-to-child co-transmission: A retrospective study in a developed country. Liver Int..

[55] Aliasi-Sinai L., Worthington T., Lange M., Kushner T. (2023). Maternal-to-Child Transmission of Hepatitis B Virus and Hepatitis Delta Virus. Clin. Liver Dis..

[56] Wu J., Wang H., Xiang Z., Jiang C., Xu Y., Zhai G., Ling Z., Chinese Consortium for the Study of Hepatitis E (CCSHE) (2024). Role of viral hepatitis in pregnancy and its triggering mechanism. J. Transl. Int. Med..

[57] Lange F., Garn J., Anagho H.A., Vondran F.W.R., von Hahn T., Pietschmann T., Carpentier A. (2023). Hepatitis D virus infection, innate immune response and antiviral treatments in stem cell-derived hepatocytes. Liver Int..

[58] Giersch K., Bhadra O.D., Volz T., Allweiss L., Riecken K., Fehse B., Lohse A.W., Petersen J., Sureau C., Urban S. (2019). Hepatitis delta virus persists during liver regeneration and is amplified through cell division both in vitro and in vivo. Gut.

[59] Cross A., Harris J.M., Arbe-Barnes E., Nixon C., Dhairyawan R., Hall A., Quaglia A., Issa F., Kennedy P.T.F., McKeating J.A. (2024). Characterisation of HBV and co-infection with HDV and HIV through spatial transcriptomics. eGastroenterology.

[60] Saez-Palma M., Buendia M., Leonel T., García-Pras E., Sanzo-Machuca A., Acera M., Pocurull Aparicio A., Battistella S., Locatelli M., Rodriguez-Tajes S. (2025). OS-029-YI Spatial profiling of HBVand HDV infection in human liver samples. J. Hepatol..

[61] Alfaiate D., Clement S., Gomes D., Goossens N., Negro F. (2020). Chronic hepatitis D and hepatocellular carcinoma: A systematic review and meta-analysis of observational studies. J. Hepatol..

[62] Lucifora J., Alfaiate D., Pons C., Michelet M., Ramirez R., Fusil F., Amirache F., Rossi A., Legrand A.F., Charles E. (2023). Hepatitis D virus interferes with hepatitis B virus RNA production via interferon-dependent and -independent mechanisms. J. Hepatol..

[63] Koffas A., Mak L.Y., Kennedy P.T.F. (2023). Hepatitis delta virus: Disease assessment and stratification. J. Viral Hepat..

[64] Pawlotsky J.M. (2024). Virological markers for clinical trials in chronic viral hepatitis. JHEP Rep..

[65] Wedemeyer H., Manns M.P. (2010). Epidemiology, pathogenesis, and management of hepatitis D: Update and challenges ahead. Nat. Rev. Gastroenterol. Hepatol..

[66] Kramvis A., Chang K.M., Dandri M., Farci P., Glebe D., Hu J., Janssen H.L.A., Lau D.T.Y., Penicaud C., Pollicino T. (2022). A roadmap for serum biomarkers for hepatitis B virus: Current status and future outlook. Nat. Rev. Gastroenterol. Hepatol..

[67] Degasperi E., Scholtes C., Testoni B., Renteria S.U., Anolli M.P., Charre C., Facchetti F., Plissonnier M.L., Sambarino D., Perbellini R. (2025). Differential HBV RNA and HBcrAg patterns in untreated patients with chronic hepatitis delta. J. Hepatol..

[68] Pearlman B. (2023). Hepatitis Delta Infection: A Clinical Review. Semin. Liver Dis..

[69] Tamura I., Kurimura O., Koda T., Ichimura H., Katayama S., Kurimura T. (1993). Risk of liver cirrhosis and hepatocellular carcinoma in subjects with hepatitis B and delta virus infection: A study from Kure, Japan. J. Gastroenterol. Hepatol..

[70] Gish R.G., Wong R.J., Di Tanna G.L., Kaushik A., Kim C., Smith N.J., Kennedy P.T.F. (2024). Association of hepatitis delta virus with liver morbidity and mortality: A systematic literature review and meta-analysis. Hepatology.

[71] Stockdale A.J., Degasperi E. (2024). HDV RNA and liver disease progression: What do we know?. Hepatology.

[72] Roulot D., Layese R., Brichler S., Ganne N., Asselah T., Zoulim F., Gordien E., Nahon P., Roudot-Thoraval F., DeltaVir and CirVir study groups (2024). Hepatitis D Virus Infection Markedly Increases the Risk of Hepatocellular Carcinoma in Patients with Viral B Cirrhosis. Clin. Gastroenterol. Hepatol..

[73] Jang T.Y., Wei Y.J., Liu T.W., Yeh M.L., Liu S.F., Hsu C.T., Hsu P.Y., Lin Y.H., Liang P.C., Hsieh M.H. (2021). Role of hepatitis D virus infection in development of hepatocellular carcinoma among chronic hepatitis B patients treated with nucleotide/nucleoside analogues. Sci. Rep..

[74] Shen C., Jiang X., Li M., Luo Y. (2023). Hepatitis Virus and Hepatocellular Carcinoma: Recent Advances. Cancers.

[75] Thiyagarajah K., Glitscher M., Peiffer K.H., Hildt E. (2025). Differential impact of hepatitis delta virus replication and expression of viral antigens on the cellular kinome profile. Cell Commun. Signal.

[76] Diaz G., Engle R.E., Tice A., Melis M., Montenegro S., Rodriguez-Canales J., Hanson J., Emmert-Buck M.R., Bock K.W., Moore I.N. (2018). Molecular Signature and Mechanisms of Hepatitis D Virus-Associated Hepatocellular Carcinoma. Mol. Cancer Res..

[77] Torrens L., Puigvehí M., Torres-Martín M., Wang H., Maeda M., Haber P.K., Leonel T., García-López M., Esteban-Fabró R., Leow W.Q. (2022). Hepatocellular Carcinoma in Mongolia Delineates Unique Molecular Traits and a Mutational Signature Associated with Environmental Agents. Clin. Cancer Res..

[78] Yu Z., Ma X., Zhang W., Chang X., An L., Niu M., Chen Y., Sun C., Yang Y. (2021). Microarray Data Mining and Preliminary Bioinformatics Analysis of Hepatitis D Virus-Associated Hepatocellular Carcinoma. Biomed. Res. Int..

[79] Zhang C., Wu S., Yang X.D., Xu H., Ma T., Zhu Q.X. (2021). Identification of Key Genes for Hepatitis Delta Virus-Related Hepatocellular Carcinoma by Bioinformatics Analysis. Turk. J. Gastroenterol..

[80] Karagas M.R., Kaldor J., Michaelis M., Muchengeti M.M., Alfaiate D., Argirion I., Chen X., Cunha C., Hantz S., Koljonen V. (2025). Carcinogenicity of hepatitis D virus, human cytomegalovirus, and Merkel cell polyomavirus. Lancet Oncol..

[81] Burm R., Maravelia P., Ahlen G., Ciesek S., Caro Perez N., Pasetto A., Urban S., Van Houtte F., Verhoye L., Wedemeyer H. (2023). Novel prime-boost immune-based therapy inhibiting both hepatitis B and D virus infections. Gut.

[82] Lu M., Liu Y., Li L., Liu X., Wu B., Wu Y. (2025). Hepatitis Vaccines: Recent Advances and Challenges. Vaccines.

[83] European Association for the Study of the Liver (2023). EASL Clinical Practice Guidelines on hepatitis delta virus. J. Hepatol..

[84] Kang C., Syed Y.Y. (2020). Bulevirtide: First Approval. Drugs.

[85] Anolli M.P., Degasperi E., Allweiss L., Sangiovanni A., Maggioni M., Scholtes C., Oberhardt V., Neumann-Haefelin C., Dandri M., Zoulim F. (2023). A 3-Year Course of Bulevirtide Monotherapy May Cure Hdv Infection in Cirrhotics. J. Hepatol..

[86] Lampertico P., Anolli M.P., Steppich K., Wedemeyer H. (2025). Bulevirtide Monotherapy or in Combination for Chronic Hepatitis Delta: 2025 Update. J. Viral Hepat..

[87] Asselah T., Chulanov V., Lampertico P., Wedemeyer H., Streinu-Cercel A., Pântea V., Lazar S., Placinta G., Gherlan G.S., Bogomolov P. (2024). Bulevirtide Combined with Pegylated Interferon for Chronic Hepatitis D. N. Engl. J. Med..

[88] Allweiss L., Cohen C., Dias J., Fumagalli V., Guo H., Harris J.M., Hu J., Iannacone M., Isogawa M., Jeng W.-J. (2024). Highlights from the 2023 International Meeting on the Molecular Biology of Hepatitis B virus. J. Gen. Virol..

[89] Asselah T., Chattergoon M.A., Jucov A., Streinu-Cercel A., Lampertico P., Wedemeyer H., Kennedy P.T., Gane E.J., Bullard B.L., Chow S. (2025). A Phase 2 Trial of Tobevibart plus Elebsiran in Hepatitis D. N. Engl. J. Med..

[90] Jucov A., Asselah T., Streinu-Cercel A., Gane E.J., Wedemeyer H., Lampertico P., Chattergoon M.A., Bullard B., Huang C., Acosta R. (2025). THU-243 SOLSTICE week 24 subgroup analysis: Impact of baseline viral parameters and cirrhosis status on virological and biochemical responses in participants with chronic hepatitis delta virus infection treated with tobevibart and elebsiran. J. Hepatol..

[91] Stern C., Loustaud-Ratti V., Yurdaydin C., Brancaccio G., Jachs M., Reiberger T., Bardou-Jacquet E., Metivier S., Alric L., Colombain L. (2025). Safety and efficacy of REP 2139-Mg in patients with HDV-related advanced liver disease in an international compassionate access program. J. Hepatol..

